# Arthroscopy‐Assisted Combined Lower Trapezius Tendon Transfer and Superior Capsule Reconstruction Using Single Allogenic Achilles Tendon for Massive Irreparable Posterosuperior Rotator Cuff Tears

**DOI:** 10.1002/atn2.70054

**Published:** 2026-06-08

**Authors:** Jung‐Taek Hwang, Hong Seuk Yang, Su‐Jung Seo

**Affiliations:** ^1^ Department of Orthopedic Surgery Chuncheon Sacred Heart Hospital, Hallym University Medical College Chuncheon‐si Republic of Korea; ^2^ Department of Anesthesiology and Pain Medicine Chuncheon Sacred Heart Hospital, Hallym University Medical College Chuncheon‐si Republic of Korea; ^3^ Department of Orthopedic Surgery Hallym University Medical College Chuncheon‐si Republic of Korea

## Abstract

Massive irreparable posterosuperior rotator cuff tears in relatively younger, active patients pose a significant clinical challenge. Superior capsular reconstruction (SCR) restores superior stability as a static stabilizer, whereas lower trapezius tendon transfer reestablishes external rotation and transverse plane balance as a dynamic stabilizer. We describe a simplified arthroscopy‐assisted technique combining lower trapezius tendon transfer and superior capsular reconstruction using a single allogenic Achilles tendon graft to achieve simultaneous restoration of static and dynamic stabilizers, and external rotation strength of the shoulder.

## Graphical abstract

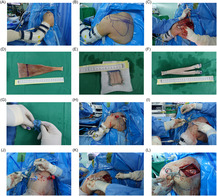

**VIDEO 1:** Arthroscopy‐assisted combined lower trapezius tendon transfer and superior capsule reconstruction using a single allogenic Achilles tendon for a massive irreparable posterosuperior rotator cuff tear in the right shoulder with the patient in the beach‐chair position. The lower trapezius tendon is harvested from the medial scapular spine and tagged with Krackow sutures. The Achilles tendon allograft is prepared for both superior capsule reconstruction and lower trapezius tendon transfer. Arthroscopic subacromial decompression and acromioplasty are performed through the lateral portal. From the lateral viewing portal, suture anchors are inserted into the glenoid through the Neviaser portal and at the cartilage‐footprint junction. The lower trapezius tendon transfer graft is introduced through the infraspinatus fossa. From the posterolateral viewing portal, both grafts are passed and fixed using a suture bridge technique through the lateral portal. Side‐to‐side sutures are placed between the superior capsule reconstruction graft and native cuff tissue, and the lower trapezius tendon is attached to the graft with the shoulder in maximal external rotation. Postoperative radiographs demonstrate reduction of superior migration of the humeral head. Video content can be viewed at https://doi.org/10.1002/atn2.70054.

Each year in the United States over 4.5 million patient visits and approximately 400,000 surgeries are attributed to rotator cuff problems.^1^ In the setting of a primary massive rotator cuff repair, the failure rate can be as high as 94%.^2^ The natural history of massive chronic irreparable rotator cuff tears involve a predictable progression to arthritic changes of the glenohumeral joint.^3^

Treatment options for irreparable posterosuperior massive rotator cuff tears include superior capsular reconstruction (SCR), lower trapezius tendon transfer (LTTT), and reverse total shoulder arthroplasty (RTSA). The surgical indication of RTSA usually includes age more than 65 years.^1^ Therefore, SCR or LTTT could be commonly used for patients younger than 65 years. SCR was introduced by Dr. Mihata for irreparable posterosuperior massive rotator cuff tears in 2012.^4^ Using autologous fascia lata or allogenous dermis, superior capsule is reconstructed as static stabilizer. LTTT was reported by Dr. Elhassan in 2016 for irreparable posterosuperior massive rotator cuff tears.^5^ Using allogenous Achilles or semitendinosus tendon, lower trapezius tendon is transferred to infraspinatus insertion as dynamic stabilizer.^6^

LTTT combined SCR could be a best option to get a more improved outcome in primary or revisional massive irreparable posterosuperior rotator cuff tears. The purpose of the present technical note is to describe an applicable treatment option to provide static and dynamic stabilizers to the affected shoulder.

## SURGICAL TECHNIQUE

The protocol in the present technical note was approved by the institutional review board of Hallym University Chuncheon Sacred Heart Hospital (2024‐10‐007). The Indications and contraindications of arthroscopy‐assisted technique combining LTTT and SCR using a single allogenic Achilles tendon graft for massive irreparable posterosuperior rotator cuff tears are the following.

### Surgical Indication

1. Irreparable posterosuperior massive rotator cuff tear including supraspinatus and infraspinatus (primary or revision cases).
2. Goutallier grade ≥2 fatty infiltration of the supraspinatus and infraspinatus.
3. Rotator cuff tear arthropathy Hamada stage ≤3.
4. Positive external rotation lag sign.
5. Preserved subscapularis and deltoid function.

### Contraindication

1. Rotator cuff tear arthropathy Hamada stage ≥4.
2. Irreparable subscapularis tendon tear.
3. Goutallier grade ≤1 fatty infiltration of the supraspinatus and infraspinatus.
4. Neurological disorder.

Age is only a relative contraindication. The surgical steps, pearls, and pitfalls are shown in Tables 1 and 2.

**TABLE 1:** Surgical Steps, Pearls, and Pitfalls

Surgical Step	Tip	Pitfall
Patient preparation and set up	1. Beach chair position, with nearly entire ipsilateral half of back exposed for approach 2. Arm traction with elbow brace or arm holder	Difficult harvesting of LTT due to inadequate position and exposure
LTT harvest	1. A 7 cm transverse incision along medial scapular spine 2. Palpation of lower trapezius insertion on the mid portion of scapular sine 3. Complete release of lower trapezius tendon from scapular spine	Care must be taken to prevent the spinal accessory nerve injury that is located 3 to 4 cm medial to the scapula.
Achilles tendon allograft preparation	1. Three sutures in a Krackow configuration are placed in the LTT allograft. 2. The folded and sutured broader portion is for SCR allograft	Inadequate dividing should be avoided. Length should be more than 200 mm. In addition, 220 mm could be needed for a large male.
Arthroscopic portals and anchor placement	1. Standard portals (anterior, posterior, lateral) and additional portals (Neviaser, Anchor suture portal, posterolateral) are used. 2. The lateral portal should be enlarged just before the allograft passage.	PassPort cannula might be an obstacle for allograft passage; therefore, it could be removed just before the passage.
Dual allograft passage	1. LTT allograft is passed through infraspinatus fossa. 2. SCR and LTT allografts with suture limbs are moved into the shoulder through the enlarged lateral portal	Back Grasper could be weak to control the allograft. Therefore, surgeon can use its thumb or index finger, additionally.
Dual allograft intraarticular attachment	1. First, glenoid tying is performed. 2. Next, medial tying is done. 3. Finally, lateral fixation of suture bridge technique is done. 4. Two lateral suture limbs of LTT graft are fixed at anterosuperior footprint.	Narrow view could make the procedure difficult. Step by step procedure is important.
Attachment of LTT to LTT allograft	1. Attachment is done at maximal external rotation. 2. Allograft trimming could be possible for adequate tension.	Take care not to cut the Krackow sutures.

LTT, lower trapezius tendon; SCR, superior capsular reconstruction.

**TABLE 2:** Advantages and Disadvantages

Advantages	Disadvantages
Easier to harvest compared with LDT. No critical neurological risks. Cost effective. More anatomic reconstruction. Better biomechanical properties than LTTT or SCR alone. Prevent synovial fluid leakage from glenohumeral joint to subacromial space which could be helpful for LTT graft healing.	Narrow view due to dual grafts. Longer operation time. Extensive arthroscopic technique.

LDT, latissimus dorsi transfer; LTTT, lower trapezius tendon transfer; SCR, superior capsular reconstruction.

### Patient Positioning

**FIGURE 1:**
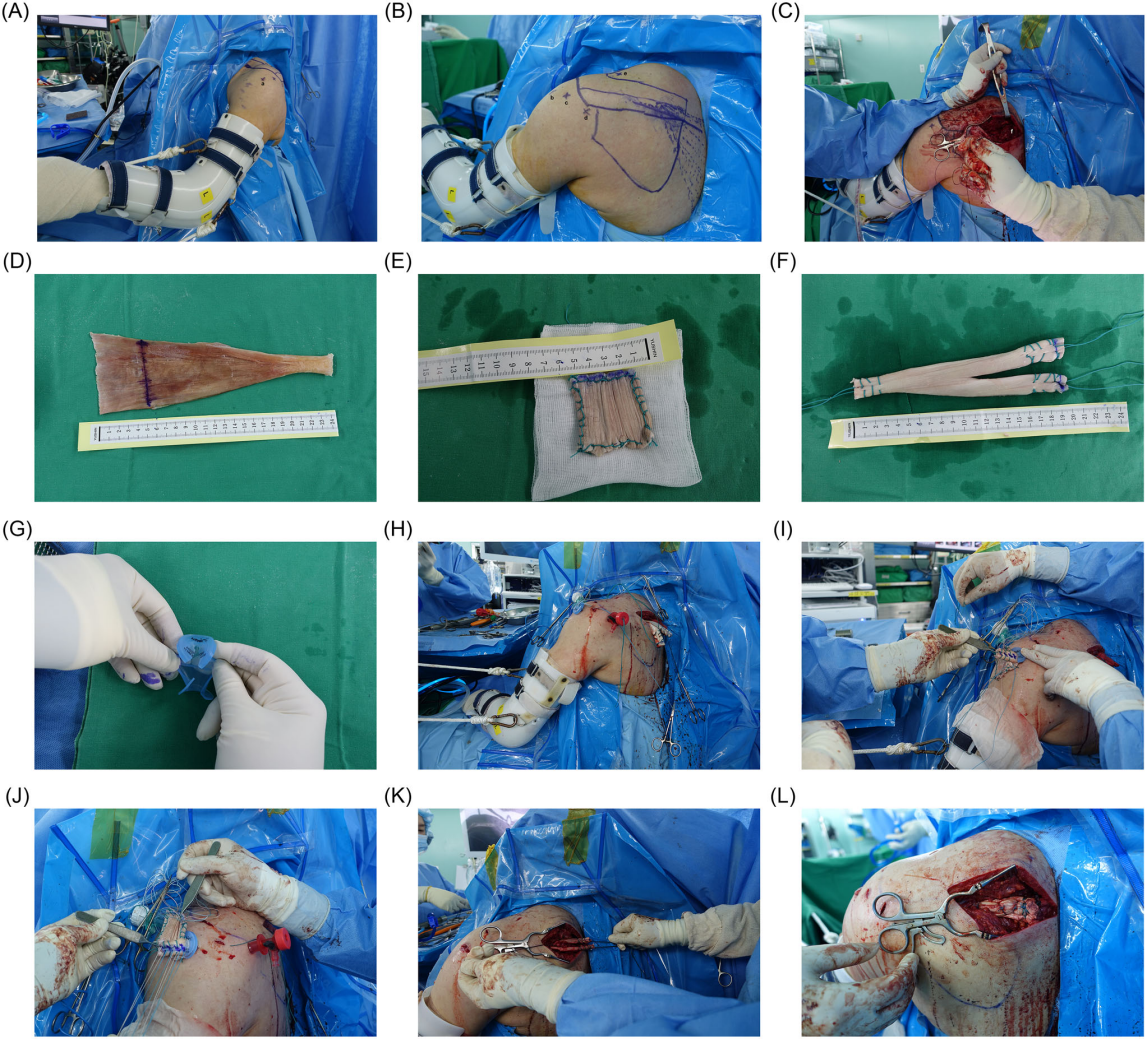
Surgical techniques of the left shoulder. (A) Beach‐chair position with elbow brace and traction. (B) A 7 cm incision along the medial scapular spine. (C) LTT harvest and tagging. (D) Single Achilles tendon allograft for dual purposes. (E) SCR graft. (F) LTT graft. (G) One sided transected PassPort cannula (Arthrex). (H) LTT graft passage. (I) Suture limbs passage through dual grafts. (J) Dual grafts passage into joint. (K) Tensioning of LTT graft in shoulder maximal external rotation. (L) Attachment of LTT graft to LTT. (a) Anterior portal. (b) Lateral portal. (c) Posterolateral portal. (d) Posterior portal. (e) Neviaser portal. (f) Harvested LTT. (LTT, lower trapezius tendon transfer; SCR, superior capsular reconstruction.)

The patient is placed in the beach chair position under general anesthesia. The traction with the elbow brace is applied and the entire ipsilateral half of back is exposed to enable an approach to the medial scapular spine for harvesting LTT (Figure 1A,B).

### Harvest of Lower Trapezius

An 8‐cm horizontal incision is performed just below the medial scapular spine over the lower trapezius tendon insertion. Meticulous dissection of subcutaneous tissue is made until the tendon is exposed. The LTT which is originated from T4 to T12 is detached from the medial scapular spine and mobilized from the middle trapezius muscle. Care should be taken to protect the spinal accessory nerve that runs 3 to 4 cm medial to the scapula. The tendinous portion of LT is sutured using No. 2 Ethibond (Ethicon, Raritan, NJ) in a Krackow configuration to facilitate later manipulation (Figure 1C).

### Graft Preparation for Combined LTTT and SCR

Allograft preparation is performed simultaneously while performing the LT harvest. A single Achilles tendon allograft is preferred for our combined LTTT and SCR. For SCR graft, the broader 5 cm portion of Achilles allograft is separated from the narrower portion. The broad portion is folded into 2 layers and then is made as a 4 × 5 cm sized two‐layered graft using the Krackow suture with No. 2 Ethibond (Ethicon, Raritan, NJ) for SCR. The remaining narrower portion is made into 3 limbed graft for LTTT. The narrower portion of this graft is made as 1 limb, and the broader portion is made as 2 limbs by dividing and suturing using No. 5 Ethibond (Ethicon, Raritan, NJ) in a Krackow configuration for later fixation (Figure 1D‐F). Eventually, the single Achilles tendon allograft is prepared to serve dual purposes.

### Arthroscopic Allograft Passage and Fixation

**FIGURE 2:**
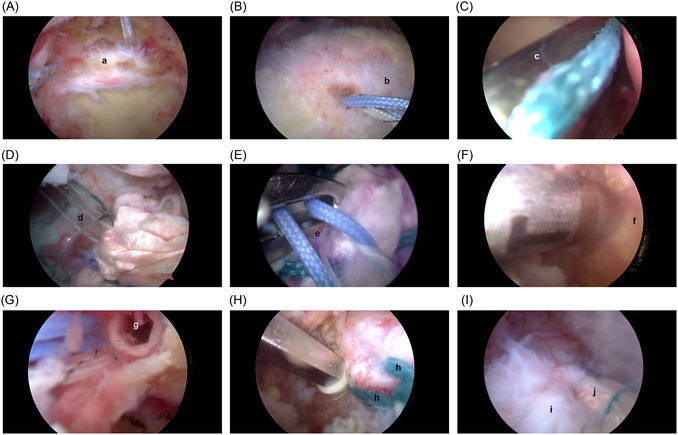
Arthroscopic views of the left shoulder in the beach‐chair position. (A) From lateral view, fixation on glenoid using Y‐knot RC suture anchor (CONMED) through Neviaser portal. (B) From lateral view, 2 anchor fixation. (B) From lateral view, anchor fixation on the junction between cartilage and footprint. (C) From lateral view, LTT graft passage using a Kelly. (D) From lateral view, tie at glenoid. (E) From posterolateral view, tie at the junction between cartilage and footprint. (F) From posterolateral view, awling on greater tuberosity for suture bridge technique. (G) From posterolateral view, SwiveLock (Arthrex) fixation on GT. (H) From posterolateral view, another SwiveLock (Arthrex) anterosuperior fixation using the Ethibond of distal LTT graft. (I) Well‐fixed SCR and LTT grafts. (a) Superior glenoid. (b) Cartilage‐footprint junction. (c) A large Kelly clamp with leading suture limbs of LTT graft. (d) Suture limbs of glenoid through anterior portal. (e) Suture limbs of cartilage‐footprint junction through lateral portal. (f) Greater tuberosity. (g) SwiveLock fixed on greater tuberosity. (h) Leading suture limbs of LTT graft. (i) SCR graft. (j) LTT graft. (LTT, lower trapezius tendon transfer; SCR, superior capsular reconstruction.)

Using standard arthroscopic portal, an examination is done. An irreparable posterosuperior massive rotator cuff tear is verified. Using Neviaser portal and posterior portal, two 2.8 mm Y‐knot RC suture anchors (CONMED, Largo, FL) are fixed on the superior glenoid. And a suture anchor portal just lateral from the lateral acromion was made, and through this portal, another 2 Y‐knot RC suture anchors are fixed on the junction of the articular cartilage and the medial aspect of the footprint on the greater tuberosity (Figure 2A,B). All the suture limbs are passed through lateral portal using a 10 mm × 4 cm PassPort cannula (Athrex, Naples, FL) which is cut longitudinally at 1 side to facilitate suture limbs to be moved from inside to outside of the cannula (Figure 1G). And then, the narrower portion of LTT allograft is located on the posterosuperior glenoid from infraspinatus space with a big Kelly clamp and the 2 limbs of sutured Ethibond (Ethicon, Raritan, NJ), and the 2 limbs is also located through the lateral portal (Figures 1H and 2C). From proximal to distal order, the SCR allograft are passed using the suture limbs and the distal suture limbs are also located at a 45° oblique fashion through the LTT allograft at the upper side of the distal portion of SCR allograft. The 2 allografts are moved inside through the lateral portal which is transversely enlarged up to 3 cm using a surgeon's thumb, index finger, or a back grasper (Athrex, Naples, FL) (Figure 1I,J). First, the sutures are tied at the superior glenoid using a sliding knot (SMC knot). Next, the remaining sutures are tied at the lateral side using the anterior, posterior, and posterolateral portals. Finally, the suture bridge technique is used for finishing lateral side fixation using two 4.75 mm Bio SwiveLock (Arthrex, Naples, FL). Then, the 2 limbs of the sutured Ethibond (Ethicon, Raritan, NJ) are fixed anterosuperior to the 2 SwiveLocks using another one (Figure 2D‐I).

### Attachment of Lower Trapezius Tendon With LTT Allograft

With the arm in maximal external rotation and 0° abduction, the 2 limbs of the LTT allograft are attached to LTT using No. 5 Ethibond (Ethicon) in the Krackow configuration (Figure 1K,L). If the distal portion of LTT allograft is too long, a little resection is possible to achieve an acceptable tension. After irrigation, wound is closed layer by layer. The patient is placed in a brace set at 30° abduction and 0° external rotation.

### Postoperative Management

The abduction brace with 0° external rotation is used for 6 weeks. From 2 to 6 weeks, assisted active forward elevation under 90° is permitted using a pulley or contralateral arm. Six weeks after the operation, assisted active exercise with a tolerable range using a stick is permitted. Ten weeks after the operation, active rotator cuff strengthening exercise is started using a yellow theraband. Sixteen weeks after the operation, the theraband exercise is continued and gradually upregulated according to the muscle strength until it will be around the strength of contralateral side.

A step‐by‐step description of the present operation is provided in Video 1.

## DISCUSSION

SCR improves superior stability and muscle balance, and decreases humeral head migration.^1^, ^6^, ^7^ It shows shoulder function improvement in short‐term follow‐up. Lower trapezius muscle shows a similar excursion to the infraspinatus muscle.^6^ Thus, LTTT can restore external rotation and transverse couple force.^1^, ^7^ Therefore, a synergistic effect can be expected if LTTT and SCR are performed simultaneously as static and dynamic stabilizers.^6^ Graft failure rate could be reduced because combined SCR might prevent synovial leakage from glenohumeral joint to subacromial space in this biologically favorable environment.^8^

One retrospective comparative study in 2022 reported that although SCR and LTT both provided improvements in overall clinical outcomes for posterosuperior irreparable rotator cuff tears with high‐grade 4 fatty infiltration in the infraspinatus, LTT was superior in terms of functional improvement, patient satisfaction, progression of arthritis, and graft integrity.^7^ In 2023, another retrospective cohort study presented that each SCR or LTTT provided improved clinical outcomes for posterosuperior irreparable rotator cuff tears. In addition, SCR led to better pain relief and restoration of forward elevation whereas LTT provided more reliable improvement in external rotation.^1^

One technical note described LTTT with autologous semitendinosus tendon and SCR with long head of biceps for massive irreparable posterosuperior rotator cuff tears. Another technical note presented LTTT combined with SCR using tibialis anterior tendon allograft. The 2 techniques attempted LTTT combined with SCR but did not provide complete SCR because of insufficient grafts.^9^, ^10^ Therefore, this arthroscopic‐assisted technique combining LTTT and SCR using a single allogenic Achilles tendon graft could provide a LTTT combined with complete SCR to achieve more improved biomechanics.

In 2023, a cadaveric study presented that SCR combined with LTTT showed improved shoulder kinematics and contact pressures in the posterosuperior massive rotator cuff tear model compared with SCR or LTTT alone.^6^ And in 2025, a case series with 15 patients showed that combined SCR using fascia lata autograft contributes to significant pain relief and functional improvements with a graft healing rate of 86.7% at a minimum 12‐month follow‐up in patients with posterosuperior irreparable massive rotator cuff tears and high‐grade fatty infiltration of the infraspinatus.^11^

In the present technique, the length of Achilles tendon allograft should be more than 200 mm because LTT allograft must be at least 150 mm. For average women, 150 mm is enough, and for average men, the length should be at least 210 mm considering the length of SCR graft as 50 mm. In a large‐sized male patient, the adequate length could be 220 mm. Achilles allografts with lengths exceeding 220 mm are available in the literature.^12^, ^13^

The described method provides a streamlined, biologically and mechanically favorable approach to manage massive irreparable posterosuperior rotator cuff tears in relatively younger and active patients, delaying or avoiding RTSA. The technique could be reproducible, cost‐effective, and may avoid the complications associated with humeral tunnels.

## DISCLOSURES

The authors (J‐T.H., H.S.Y., S‐J.S.) declare that they have no known competing financial interests or personal relationships that could have appeared to influence the work reported in this article.

## FUNDING

This research was supported by Hallym University Medical Center Research Fund which was received by Jung‐Taek Hwang.
